# Noc Corrals Migration of FtsZ Protofilaments during Cytokinesis in Bacillus subtilis

**DOI:** 10.1128/mBio.02964-20

**Published:** 2021-02-02

**Authors:** Yuanchen Yu, Jinsheng Zhou, Frederico J. Gueiros-Filho, Daniel B. Kearns, Stephen C. Jacobson

**Affiliations:** a Department of Chemistry, Indiana University, Bloomington, Indiana, USA; b Department of Biology, Indiana University, Bloomington, Indiana, USA; c Department of Biochemistry, Universidade de São Paulo, São Paulo, Brazil; University of Michigan—Ann Arbor

**Keywords:** FtsZ, Noc, microfluidics, growth, cell division, ZapA, binary fission, nucleoid occlusion

## Abstract

Bacteria that divide by binary fission form FtsZ rings at the geometric midpoint of the cell between the bulk of the replicated nucleoids. In Bacillus subtilis, the DNA- and membrane-binding Noc protein is thought to participate in nucleoid occlusion by preventing FtsZ rings from forming over the chromosome. To explore the role of Noc, we used time-lapse fluorescence microscopy to monitor FtsZ and the nucleoid of cells growing in microfluidic channels. Our data show that Noc does not prevent *de novo* FtsZ ring formation over the chromosome nor does Noc control cell division site selection. Instead, Noc corrals FtsZ at the cytokinetic ring and reduces migration of protofilaments over the chromosome to the future site of cell division. Moreover, we show that FtsZ protofilaments travel due to a local reduction in ZapA association, and the diffuse FtsZ rings observed in the Noc mutant can be suppressed by ZapA overexpression. Thus, Noc sterically hinders FtsZ migration away from the Z-ring during cytokinesis and retains FtsZ at the postdivisional polar site for full disassembly by the Min system.

## Abstract (precis)

In bacteria, a condensed structure of FtsZ (Z-ring) recruits cell division machinery at the midcell, and Z-ring formation is discouraged over the chromosome by a poorly understood phenomenon called nucleoid occlusion. In B. subtilis, nucleoid occlusion has been reported to be mediated, at least in part, by the DNA-membrane bridging protein, Noc.

## INTRODUCTION

Many bacteria grow and divide by a process called binary fission, in which cells increase in biomass and divide into two apparently identical siblings. Prior to division, chromosomes compacted into structures called nucleoids are replicated and segregated to become evenly distributed in the cell (1–4). Next, the early cell division scaffolding protein FtsZ forms an intense ring-like structure in a space between the replicated nucleoids and recruits the machinery for cell envelope biosynthesis (5–8). FtsZ is thought to become concentrated in the interchromosome space, at least in part by a process called nucleoid occlusion. Nucleoid occlusion is the idea that the presence of the nucleoid inhibits the formation of FtsZ at places along the cell length where the concentration of the chromosomal DNA is greatest (9–12). In so doing, the nucleoid helps guide the division machinery between the replicated chromosomes such that subsequent division will not only perform cytokinesis but also ensure that each daughter receives one copy of the genome (13, 14). The molecular mechanisms underpinning the phenomenon of nucleoid occlusion are poorly understood.

In the Gram-positive bacterium Bacillus subtilis, nucleoid occlusion is believed to be mediated, at least in part, by the protein Noc. The *noc* gene was discovered serendipitously when double mutants with the *min* system caused a temperature-sensitive defect in cell division (15). Mutation of *noc* alone did not affect growth but resulted in spiral-like intermediates of FtsZ that were observed over the nucleoid, and treatment of a *noc* mutant with a DNA-damaging agent resulted in a lethal bisection of trapped chromosomes (15–17). Noc is a ParB-like DNA binding protein that binds to many sites across the chromosome and has an amphipathic helix that promotes membrane association (18, 19). No direct interaction has been reported between Noc and FtsZ, and Noc has been proposed to act passively by bringing regions of the chromosome to the membrane and sterically excluding membrane-proximal FtsZ structures from forming (19). The Noc model is dissimilar to the proposed mechanism of SlmA, an unrelated DNA binding protein that mediates nucleoid occlusion in Escherichia coli (20). SlmA has been shown to interact with FtsZ directly and promote protofilament disassembly and/or misalignment *in vitro* (21–25). Thus, nucleoid occlusion in E. coli seems to be an active mechanism, whereas nucleoid occlusion in B. subtilis is thought to be passive.

Here, we used a programmable microfluidic device with an integrated microchannel array (26, 27) to monitor B. subtilis FtsZ dynamics in the presence and absence of Noc during chemostatic growth. Our data suggest that rather than functioning as an inhibitor of Z-ring formation, Noc functions as an inhibitor of FtsZ migration. We observed that mutation of Noc resulted in a decondensed spiral-like intermediate of FtsZ over nucleoids, consistent with previous reports. However, the decondensed spiral structures did not appear to have formed *de novo* but rather split from preexisting FtsZ rings and migrated to the future site of cell division. We further show that the spiral-like intermediates are locally depleted for association with the Z-ring-stabilizing protein ZapA and that artificial overexpression of ZapA recondenses FtsZ into a tight ring in the absence of Noc. Thus, Noc appears to corral the FtsZ ring by preventing the redistribution of decondensed protofilaments in a manner analogous to the role of septins in eukaryotic cytokinesis (28–31). We suggest that Noc recruits the nucleoid to the membrane and sterically confines FtsZ, both during cytokinesis and after it is complete.

## RESULTS

### Noc prevents FtsZ from traveling over the chromosome.

**FIG 1:**
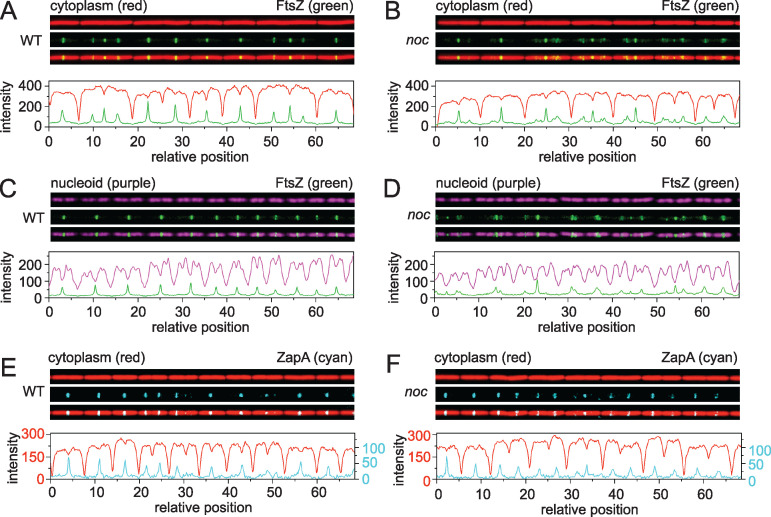
Microfluidic analysis of growth, division, and chromosome segregation in the wild type and *noc* mutants. Fluorescence microscopy of the wild type (A, C, E) and *noc* mutants (B, D, F) growing at steady state in a microfluidic channel. (A and B) Fluorescence microscopy of the wild type (DK5133) (A) and the *noc* mutant (DK5820) (B). Cytoplasmic mCherry false colored red (top), mNeongreen-FtsZ false colored green (middle), and an overlay of the two colors (bottom) are shown. Graphs are a quantitative analysis of mCherry fluorescence intensity (red line) and mNeongreen fluorescence intensity (green line) to match the fluorescence images immediately above. (C and D) Fluorescence microscopy of the wild type (DK5712) (C) and a *noc* mutant (DK6372) (D). Chromosomal HBsu-mCherry false colored purple (top), mNeongreen-FtsZ false colored green (middle), and an overlay of the two colors (bottom) are shown. Graphs are a quantitative analysis of mCherry fluorescence intensity (purple) and mNeongreen fluorescence intensity (green) to match the fluorescence images immediately above. (E and F) Fluorescence microscopy of the wild type (DK 8138) (E) and a *noc* mutant (DK8172) (F). Cytoplasmic mCherry false colored red (top), ZapA-mNeongreen false colored cyan (middle), and an overlay of the two colors (bottom) are shown. Graphs are a quantitative analysis of mCherry fluorescence intensity (red) and mNeongreen fluorescence intensity (cyan) to match the fluorescence images immediately above. For panels E and F, two different *y* axes are used due to lower fluorescence intensity from the ZapA-mNeongreen construct. The left axis corresponds to the mCherry signal (red), and the right axis corresponds to the ZapA-mNeongreen signal (cyan). All images are reproduced at the same magnification.

**FIG 2:**
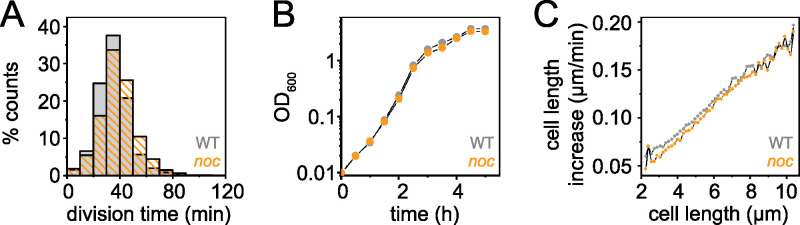
Cells mutated for Noc grow like the wild type. (A) Histogram of the division time of individual cells of the wild type (gray) and a *noc* mutant (orange) measured by microscopic analysis. Division events were defined by a local 20% decrease in mCherry (cytoplasmic) fluorescence intensity below a threshold value, and the division time was the time between two consecutive division events. More than 2,800 division events were counted per data set. (B) Growth curve of the wild type (gray) and a *noc* mutant (orange) growing in highly agitated LB broth at 37°C. Optical density was measured with a spectrophotometer at a 600-nm wavelength. (C) Increase in cell length for the wild type (gray) and the *noc* mutant (orange) was measured as the rate at which the cell poles moved apart from one another relative to the current cell length. Each panel was generated using strains DK5133 (WT) and DK5820 (*noc*).

In B. subtilis, the protein Noc is thought to function in nucleoid occlusion by preventing FtsZ-ring formation and subsequent cell division from occurring over the chromosome. We used a microfluidic platform with microchannel arrays to quantitatively study the role of Noc on bacterial cell division during chemostatic growth. Fluorescence images of cells expressing cytoplasmic red fluorescent protein were captured every 2 min after cells entered steady-state growth, and cell division was defined as a 20% decrease in fluorescence intensity along the longitudinal axis of the cell (Fig. 1A). Microfluidic analysis showed that most of the cell division events occurred in the middle of the *noc* mutant (Fig. 1B), with an average division time similar to that of the wild type (Fig. 2A). The growth rate measured by optical density in liquid culture (Fig. 2B) and cellular elongation rate per cell length (Fig. 2C) of the *noc* mutant were also similar to that of the wild type. We conclude that disruption of *noc* had minimal effects on the overall growth and division of the cell.

**FIG 3:**
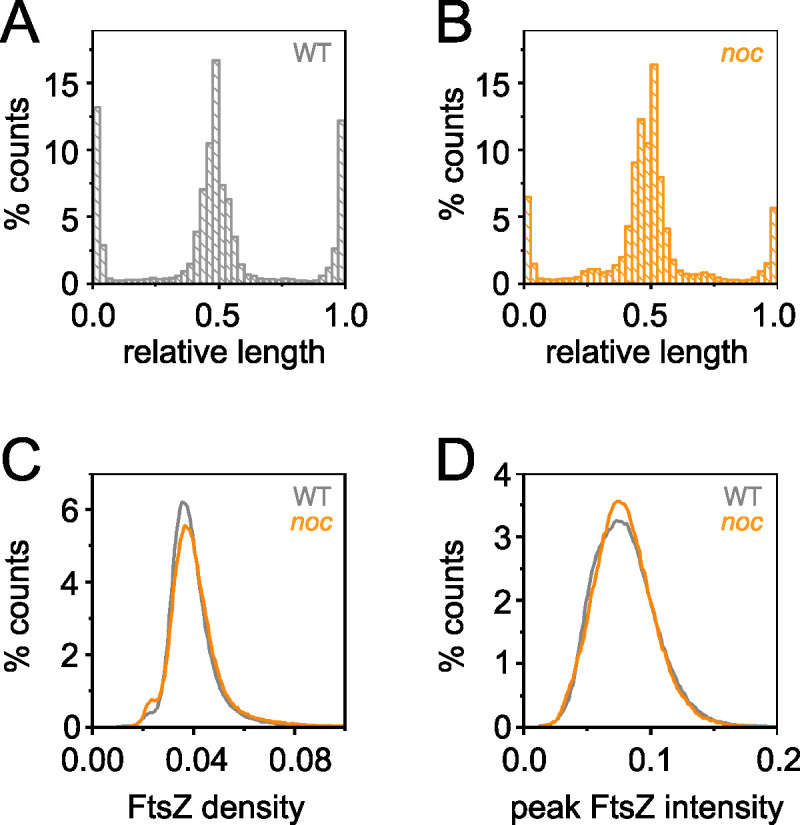
Peak FtsZ intensity at locations other than the poles and midcell increased in the *noc* mutant. (A) Histogram of the peak FtsZ intensity along the relative cell length of the wild type (gray). Relative lengths are 0 and 1.0 for the cell poles and 0.5 for the midcell. (B) Histogram of the peak FtsZ intensity along the relative cell length of the *noc* mutant (orange). (C) Histogram of FtsZ density in individual cells measured by dividing the total FtsZ fluorescence intensity (see Fig. S2A in the supplemental material) by cell length (Fig. S2B) for the wild type (gray) and *noc* mutant (orange). (D) Histogram of peak FtsZ fluorescence intensity in individual cells for the wild type (gray) and *noc* mutant (orange). Data for each panel were generated by a line scan along the longitudinal axis of the cell to determine the location of peak fluorescence intensity for each frame during time-lapse microscopy. Data were taken from 13,000 wild-type cells (DK5133) for >420,000 measurements and 5,000 *noc* mutant cells (DK5820) for >170,000 measurements.

**FIG 4:**
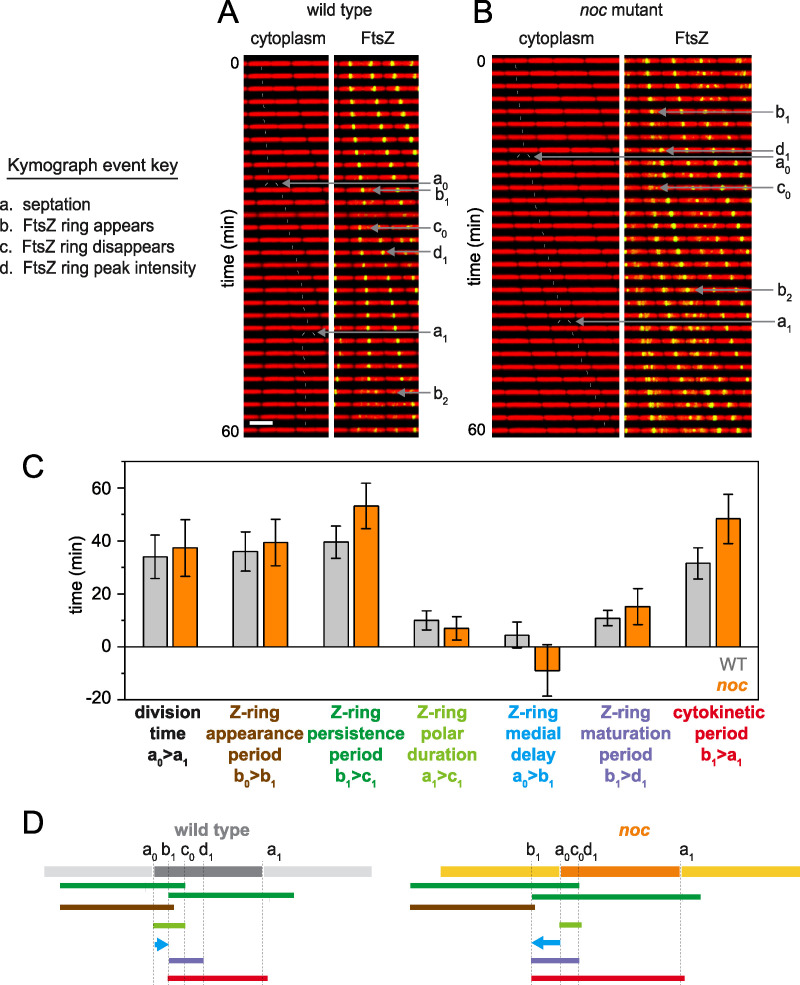
In *noc* mutants, a subset of FtsZ disassociates from the Z-ring and travels to the future site of cell division, which causes the Z-ring medial delay time to become negative. (A and B) Kymograph analysis of the wild type (A) and *noc* mutant (B) grown in a microfluidic channel and imaged every 2 min. Cytoplasmic mCherry signal is false colored red (left) and overlaid with mNeongreen-FtsZ that is false colored green (right). Events necessary for defining division parameters are indicated and labeled as follows: a, septation; b, appearance of a nascent Z-ring; c, disappearance of a Z-ring; d, FtsZ peak intensity achieved. Each event designation is given a number, as follows: 0 for the preceding generation, 1 for the current generation, and 2 for the subsequent generation. Thin white lines are included to indicate cell tracking and lineage analysis. Scale bar, 5 μm (all panels). (C) Graphs of 100 manually tracked cell division cycles for the wild type (gray) and *noc* mutant (orange) presented as bars for average values and whiskers for standard deviation for the following parameters: division time, time between septation events (between consecutive “a” events); Z-ring appearance period, time between the appearance of one Z-ring and another (between consecutive “b” events); Z-ring persistence period, time between the appearance of a Z-ring and the disappearance of that Z-ring (between consecutive “b” and “c” events); Z-ring polar duration, time between a septation event and the disappearance of the Z-ring resulting from that septation event (between consecutive “a” and “c” events); Z-ring medial delay, time between a septation event and the appearance of a Z-ring that will eventually give rise to the next medial division event (between an “a” event and a “b” event that will give rise to the next round of septation); Z-ring maturation period, time between Z-ring appearance and when that Z-ring achieves peak local intensity (between consecutive “b” and “d” events); cytokinetic period, time between Z-ring appearance and septation directed by that Z-ring (between a “b” event and the “a” event that is caused by that particular Z-ring). In the case of the *noc* mutant, the Z-ring that will form the future division site first appears by splitting from the preexistent Z-ring, and FtsZ appearance is defined as the time at which the Z-ring visibly splits from its progenitor. Note that the Z-ring medial delay of the *noc* mutant was negative on average because the medial Z-ring that would eventually promote cell division was formed in the preceding generation. Histograms for each bar are presented in Fig. S2C to H in the supplemental material. (D) Timelines of the various events indicated in the bar graph, color coded to match the indicated parameter of like color above and annotated with relevant events marked by the defining letters. Each panel was generated using strains DK5133 (WT) and DK5820 (*noc*).

Nucleoid occlusion is thought to be mediated by preventing FtsZ ring formation in the vicinity of the chromosomes. To investigate the localization of the Z-ring, mNeongreen was fused to the N terminus of FtsZ in merodiploidy at the native site (32, 33) (Fig. S1 in the supplemental material), and FtsZ dynamics were tracked in the wild type (Fig. 1A; see Movie S1) and the *noc* mutant (Fig. 1B; Movie S2). In the wild type, FtsZ appeared as a tight or “condensed” band, with peak intensity localized primarily to the midcell and cell poles (Fig. 3A), and while this localization was also largely true for the *noc* mutant, FtsZ peak intensity was observed at other locations with increased frequency (Fig. 3B). Moreover, the FtsZ rings observed in the *noc* mutant often appeared loose or “decondensed” in that they were wider and more diffuse (Fig. 1B). Kymograph analysis showed that in the wild type, FtsZ rings appeared as intense bands that transiently persisted at the cell pole after cytokinesis, before disappearing and reappearing at the nascent midcell (Fig. 4A). In the *noc* mutant, however, a subpopulation of FtsZ appeared to split from the primary ring and migrate toward the next midcell as a spiral-like intermediate (Fig. 4B). We infer that migration of a decondensed FtsZ-ring in the *noc* mutant was responsible for the increase in frequency at which peak intensity was observed at positions other than the midcell and poles. We conclude that Noc does not prevent *de novo* formation of Z-rings over the nucleoid. Rather, Noc stabilizes FtsZ rings by inhibiting a portion of FtsZ from migrating away from the site of cytokinesis along the length of the cell, likely over the top of the chromosome.

10.1128/mBio.02964-20.1 FIG S1 mNeongreen-FtsZ is expressed in merodiploidy with the wild-type copy. Western blot analysis of the wild type (DK1042) and the mNeongreen-FtsZ-expressing DK5092. Top panel, Western blot of cell lysates from the indicated strains probed with anti-FtsZ primary antibody (generous gift of Petra Levin, Washington University). Bottom panel, Western blot analysis of cell lysates from the indicated strains probed with anti-SigA primary antibody (generous gift of Masaya Fujita, University of Houston). Download FIG S1, EPS file, 1.8 MB. Copyright © 2021 Yu et al. 2021 Yu et al. This content is distributed under the terms of the Creative Commons Attribution 4.0 International license.

10.1128/mBio.02964-20.4 MOVIE S1 Wild-type growth in microfluidic channels with fluorescent FtsZ. Constitutive cytoplasmic mCherry is false colored red, and mNeongreen-FtsZ is false colored green. Strain DK5133. Movies are 100 frames over 200 min at a rate of 1 frame/2 min. Download Movie S1, AVI file, 1.7 MB. Copyright © 2021 Yu et al. 2021 Yu et al. This content is distributed under the terms of the Creative Commons Attribution 4.0 International license.

10.1128/mBio.02964-20.5 MOVIE S2 *noc* mutant growth in microfluidic channels with fluorescent FtsZ. Constitutive cytoplasmic mCherry is false colored red, and mNeongreen-FtsZ is false colored green. Strain DK5820. Movies are 100 frames over 200 min at a rate of 1 frame/2 min. Download Movie S2, AVI file, 1.8 MB. Copyright © 2021 Yu et al. 2021 Yu et al. This content is distributed under the terms of the Creative Commons Attribution 4.0 International license.

10.1128/mBio.02964-20.2 FIG S2 Quantitative analysis of micrographs. (A) Histogram of total FtsZ fluorescence intensity in individual cells for the wild type (gray) and a *noc* mutant (orange). For each frame, a line scan through the longitudinal axis of the cell was performed, and total FtsZ fluorescence intensity was measured by integrating the area under the line scan. (B) Histogram of individual cell lengths for the wild type (gray) and a *noc* mutant (orange). Data in panels A and B were taken from 13,000 wild-type cells (DK5133) for >420,000 measurements and 5,000 *noc* mutant cells (DK5820) for >170,000 measurements. (C to H) One hundred cells each of the wild type (DK5133, gray) and a *noc* mutant (DK5820, orange) were manually tracked and measured through a cell division cycle to generate the data for each panel. (C) Histogram of the Z-ring appearance period, defined as the time between the appearance of one Z-ring and that of the next Z-ring. Note that in the case of the *noc* mutant, the Z-ring that will form the future division site first appears by splitting from the preexisting Z-ring. Thus, for the *noc* mutant, the FtsZ appearance event was defined as the time at which the Z-ring visibly splits from its progenitor. (D) Histogram of the Z-ring persistence period, defined by the time between the appearance of a Z-ring and the disappearance of that Z-ring. (E) Histogram of the Z-ring polar duration, defined as the time between a septation event and the disappearance of the Z-ring responsible for that septation event. (F) Histogram of the Z-ring medial delay, defined as the time between a septation event and the appearance of a Z-ring that will eventually give rise to the next medial division event. Note that the Z-ring medial delay of the *noc* mutant was negative on average because the medial Z-ring that would eventually promote cell division was formed by splitting from the primary focus in the preceding generation. (G) Histogram of the Z-ring maturation period, defined as the time between Z-ring appearance and when that Z-ring achieved peak local intensity. (H) Histogram of the cytokinetic period, defined as the time between Z-ring appearance and septation directed by that Z-ring. (I) Histogram of the segregation time of individual chromosomes of the wild type (DK5712, gray) and a *noc* mutant (DK6372, orange) measured by microscopic analysis. Chromosome segregation events were defined by a local 40% decrease in HBsu-mCherry fluorescence intensity below a threshold value. More than 300 division events were counted per data set. (J) Histogram of ZapA density in individual cells measured by dividing the total ZapA-mNeongreen fluorescence intensity by cell length for the wild type (DK8138, gray) and a *noc* mutant (DK8172, orange). Download FIG S2, EPS file, 2.0 MB. Copyright © 2021 Yu et al. 2021 Yu et al. This content is distributed under the terms of the Creative Commons Attribution 4.0 International license.

**FIG 5:**
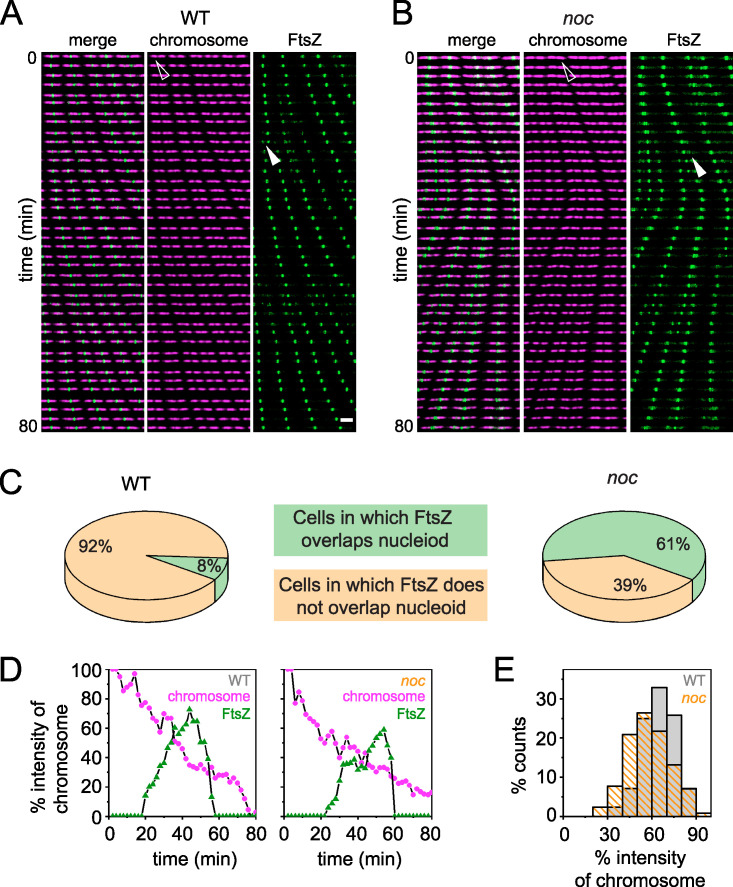
The FtsZ ring does not form in the chromosome gap earlier in the *noc* mutant. (A and B) Kymograph analysis of the wild type (A) and a *noc* mutant (B) grown in a microfluidic channel and imaged every 2 min. Chromosomal HBsu-mCherry is false colored purple, and mNeongreen-FtsZ is false colored green. All images reproduced at the same magnification. Left, color overlay; center, HBsu-mCherry; right, mNeongreen-FtsZ. Open triangle indicates the nucleoid intensity monitored in the corresponding graphs in panel D. Closed triangle indicates the FtsZ intensity monitored in the corresponding graphs in panel D. Scale bar, 5 μm (all panels). (C) Pie charts indicate the percentage of cells in which FtsZ rings are found to overlap (green) or not overlap (tan) with the bulk of the nucleoid mass in the wild type and the *noc* mutant. Two hundred cells were counted for each background. (D) HBsu-mCherry (chromosome, purple) and mNeongreen-FtsZ (FtsZ, green) intensities at midchromosome over time. Both values were normalized by the maximum intensity of the nucleoid to minimize the intensity differential across different time points. Left panel, a representative result of a single wild-type cell (triangles in panel A); right panel, a representative result of a single *noc* mutant cell (triangles in panel B). (E) Histogram of the chromosome intensity at which FtsZ first localized precisely at the newly formed chromosome gap. Data are taken from 200 cells, similar to data in panel D. Each panel was generated using strains DK5712 (WT) and DK6372 (*noc*).

To determine Z-ring localization relative to the chromosomes, the nucleoid was fluorescently labeled by fusion of the fluorescent protein mCherry to the nucleoid binding protein HBsu and incorporated in merodiploidy at an ectopic site (4, 34). During growth of the wild type, nucleoids appeared as intense fluorescence in the cytoplasm and were sometimes observed to form bi-lobed masses indicative of partial replication and segregation (Fig. 1C and 5A**;** Movie S3). Moreover, FtsZ rings formed preferentially between the lobes of the partially replicated nucleoid over the low-density interchromosome region (Fig. 1C and 5A**;** Movie S3). FtsZ rings still concentrated predominantly between chromosomes in the *noc* mutant (Fig. 1D and 5B**;** Movie S4), but spiral-like FtsZ structures were also observed over the nucleoid mass (Fig. 1D and 5B**;** Movie S4). Kymograph analysis indicated that 61% of the cells had diffuse FtsZ foci migrate from the current site of cytokinesis to the future division site by traveling over the nucleoid, compared to only 8% in the wild type (Fig. 5C). We conclude that during cytokinesis, a portion of FtsZ splits from the primary ring and migrates atop the chromosome to coalesce at the next midcell position and Noc reduces the frequency of this event. Thus, Noc does not prevent the *de novo* formation of FtsZ foci over the nucleoid but rather corrals FtsZ at its present location.

10.1128/mBio.02964-20.6 MOVIE S3 Wild-type chromosome segregation in microfluidic channels with fluorescent FtsZ. HBsu-mCherry is false colored purple, and mNeongreen-FtsZ is false colored green. Strain DK5712. Movies are 100 frames over 200 min at a rate of 1 frame/2 min. Download Movie S3, AVI file, 1.6 MB. Copyright © 2021 Yu et al. 2021 Yu et al. This content is distributed under the terms of the Creative Commons Attribution 4.0 International license.

10.1128/mBio.02964-20.7 MOVIE S4 *noc* mutant chromosome segregation in microfluidic channels with fluorescent FtsZ. HBsu-mCherry is false colored purple, and mNeongreen-FtsZ is false colored green. Strain DK6372. Movies are 100 frames over 200 min at a rate of 1 frame/2 min. Download Movie S4, AVI file, 1.5 MB. Copyright © 2021 Yu et al. 2021 Yu et al. This content is distributed under the terms of the Creative Commons Attribution 4.0 International license.

### Corralling by Noc alters FtsZ dynamics but has a neutral outcome on division.

Overexpression of FtsZ is reported to result in decondensed spiral-like FtsZ structures that travel in a manner similar to those observed in the *noc* mutant (35–38). Thus, one way in which the *noc* mutant might give rise to traveling foci is if it exhibited an increase in FtsZ expression. The total amount of FtsZ per cell appeared slightly higher in the *noc* mutant than in the wild type, as measured by mNeongreen-FtsZ fluorescence intensity (Fig. S2A), but on average, the *noc* mutant cells were also slightly longer (Fig. S2B). When length differences were factored in, the FtsZ intensity per unit length was similar in both cell types (Fig. 3C). Furthermore, both the wild type and the *noc* mutant showed similar local maxima for FtsZ peak intensity at the division site (Fig. 3D). We conclude that the absence of the Noc protein did not affect the FtsZ concentration nor did its absence alter the ability of FtsZ rings to reach peak local intensity. We infer that the absence of Noc leads to alterations in Z-ring dynamics.

To explore Z-ring dynamics, 100 cells each were chosen at random from the wild type and *noc* mutant and tracked through the course of division (27). Four events were sequentially measured, i.e., the appearance of an FtsZ ring, accumulation of FtsZ to peak intensity, cell division, and disappearance of the FtsZ ring, and used to calculate seven division parameters. Four of the seven parameters were similar for both cell types, and three were different (Fig. 4C**;** Fig. S2C to H). For example, the FtsZ persistence time, defined as the time between the appearance and disappearance of an FtsZ ring, was longer in the *noc* mutant (Fig. 4C and D**;** Fig. S2D). The longer persistence time was not due to a decreased rate of polar Z-ring disassembly (Fig. 4C and D**;** Fig. S2E), as had been observed with cells mutated for the Min system (27). Instead, the medial delay time, defined as the time between cell division and appearance of the next Z-ring, was negative, as future rings were formed by splitting from a Z-ring soon after coalescence (Fig. 4C and D; Fig. S2F). The *noc* mutant also experienced a net increase in the cytokinetic period, defined as the time between Z-ring appearance and the cell division event mediated by that Z-ring (Fig. 4C and D; Fig. S2H), again largely due to the reduction in the medial delay. Thus, FtsZ forms a ring at the midcell earlier in the *noc* mutant, but faster formation did not result in faster division, likely because cytokinesis is limited by the recycling of other divisome components.

The negative medial delay time in the *noc* mutant might suggest that FtsZ finds the interchromosome region and future site of cell division earlier than the wild type. To investigate this hypothesis, we tracked chromosomes and FtsZ foci simultaneously. We defined the chromosome segregation time as the time needed for one chromosome density to become two, as measured by a local 40% decrease in HBsu-mCherry fluorescence intensity in the interchromosome region. The chromosome segregation time was similar in both the wild type and *noc* mutant, indicating that Noc did not have a major role in either genome replication or separation (Fig. S2I). In the wild type, FtsZ rings appeared in the interchromosome region when the remaining chromosome intensity dropped to 64% ± 11%, and FtsZ rings appeared in the interchromosome region in the *noc* mutant at approximately the same chromosome intensity (Fig. 5D and E). Thus, in the absence of Noc, the decondensed spiral-like FtsZ intermediate departs from a condensed Z-ring and travels over top of the chromosome but otherwise forms a new Z-ring at the same position as the wild type. Thus, traveling FtsZ appeared to be a neutral alternative to full FtsZ ring disassembly and reassembly *de novo* at the midcell.

### ZapA overexpression condenses Z-rings in the *noc* mutant.

In the *noc* mutant, the FtsZ ring appeared decondensed insofar as it appeared to travel in a loose helical structure across the nucleoid. FtsZ polymerizes as treadmilling protofilaments which, in turn, are condensed into tight Z-rings by a protofilament bundling protein called ZapA (39–42). FtsZ might appear to be decondensed in the *noc* mutant, if the traveling focus had less ZapA than the condensed medial or postdivisional polar Z-rings. To investigate ZapA localization, mNeongreen was fused to the C terminus of ZapA at the native site in the chromosome, and dynamics were tracked in the wild type and the *noc* mutant. In the wild type, ZapA localized to the midcell and cell poles after division, much like FtsZ (Fig. 1E**;** Movie S5). In the *noc* mutant, ZapA also localized to the midcell and cell poles after division, but unlike FtsZ, ZapA did not also localize in a decondensed helical pattern (Fig. 1F**;** Movie S6). Kymograph analysis indicated that the dynamics of ZapA were similar in both the wild type and the *noc* mutant (Fig. S3), and again unlike FtsZ, the ZapA foci did not appear to travel over the chromosome in the absence of Noc (Fig. 4B). Failure of ZapA to travel with FtsZ was not due to a reduction in ZapA levels, as the amount of ZapA-mNeongreen fluorescence per unit length was similar in both the wild type and the *noc* mutant (Fig. S2J). We conclude that ZapA and FtsZ do not strictly colocalize such that in the absence of Noc, decondensed FtsZ foci that are ZapA deficient travel over the top of the chromosome.

10.1128/mBio.02964-20.3 FIG S3 In the *noc* mutants, ZapA does not migrate to the future division size with FtsZ. Kymograph analysis of the wild type (DK8138) (A) and a *noc* mutant (DK8172) (B). Cytoplasmic mCherry signal is false colored red, and ZapA-mNeongreen intensity is false colored cyan. To assemble a kymograph, a single microfluidic channel is imaged at 2-min intervals. All images were reproduced at the same magnification. Download FIG S3, EPS file, 2.8 MB. Copyright © 2021 Yu et al. 2021 Yu et al. This content is distributed under the terms of the Creative Commons Attribution 4.0 International license.

10.1128/mBio.02964-20.8 MOVIE S5 Wild-type growth in microfluidic channels with fluorescent ZapA. Constitutive cytoplasmic mCherry is false colored red, and ZapA-mNeongreen is false colored cyan. Strain DK8138. Movies are 100 frames over 200 min at a rate of 1 frame/2 min. Download Movie S5, AVI file, 1.0 MB. Copyright © 2021 Yu et al. 2021 Yu et al. This content is distributed under the terms of the Creative Commons Attribution 4.0 International license.

10.1128/mBio.02964-20.9 MOVIE S6 *noc* mutant growth in microfluidic channels with fluorescent ZapA. Constitutive cytoplasmic mCherry is false colored red, and ZapA-mNeongreen is false colored cyan. Strain DK8172. Movies are 100 frames over 200 min at a rate of 1 frame/2 min. Download Movie S6, AVI file, 0.9 MB. Copyright © 2021 Yu et al. 2021 Yu et al. This content is distributed under the terms of the Creative Commons Attribution 4.0 International license.

**FIG 6:**
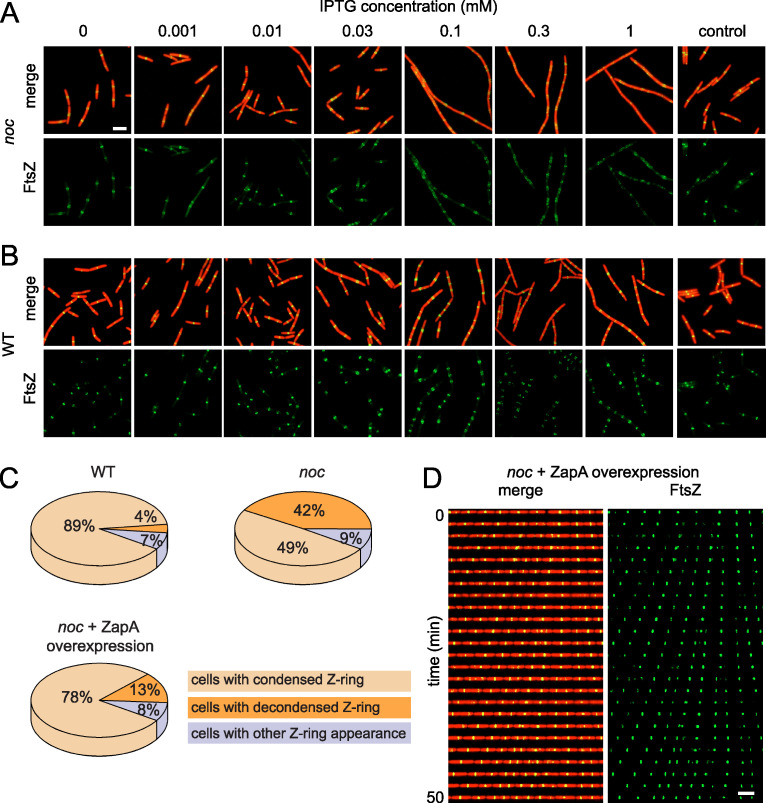
Overexpression of ZapA stabilizes FtsZ rings in a *noc* mutant. (A and B) Fluorescence microscopy of a *noc* mutant (DK8144) (A) and the wild type (DK8139) (B) containing an IPTG-inducible copy of ZapA grown in broth culture containing the indicated amount of IPTG. A *noc* mutant (DK8170) and the wild type (DK8162) lacking the inducible ZapA construct were used as controls, respectively. Constitutively expressed cytoplasmic mRFPmars is false colored red, and mNeongreen-FtsZ is false colored green. Scale bar, 5 μm (all panels). (C) Pie charts indicate the percentage of cells for each genotype that exhibited condensed Z-rings (tan), decondensed Z-rings (orange), and Z-rings with other patterns (lavender) in the indicated backgrounds. Two hundred cells each for the wild type (DK8162), *noc* mutant (DK8170), and *noc* mutant in which ZapA was induced with 0.01 mM IPTG (DK8144) were analyzed to generate each graph, respectively. (D). Kymograph analysis of a *noc* mutant (DK8144) in which ZapA was induced with 0.01 mM IPTG. Constitutively expressed cytoplasmic mRFPmars is false colored red, and mNeongreen-FtsZ is false colored green. To assemble a kymograph, a single microfluidic channel was imaged at 2-min intervals. Scale bar, 5 μm (both panels).

If decondensed, traveling FtsZ rings were due to a local reduction in ZapA association, we hypothesized that Z-rings of the *noc* mutant might be recondensed into a tight structure by ZapA overexpression. ZapA expression was controlled by cloning the *zapA* gene under the regulation of the IPTG (isopropyl-β-d-1-thiogalactopyranoside)-inducible *P_hyspank_* promoter and inserted as a merodiploid at the ectopic *amyE* site of a *noc* mutant that expressed both cytoplasmic mRFPmars and mNeongreen-FtsZ. In the absence of IPTG, the cells exhibited decondensed Z-rings consistent with the *noc* mutant phenotype (Fig. 6A). As the amount of IPTG was increased to 0.01 mM, recondensation of FtsZ occurred such that the frequency of spiral-like FtsZ structures decreased and tight Z-rings resembling that of the wild type were observed (Fig. 6A). At IPTG concentrations above 0.01 mM, however, decondensed Z-rings reappeared, and cell division was abolished altogether at the highest level of induction (Fig. 6A). An excess of IPTG also resulted in decondensed Z-rings in the wild type but did not appear to inhibit cell division as severely as when Noc was absent (Fig. 6B). We infer that the *noc* mutant is sensitized to overexpression of ZapA but that the decondensed spiral-like Z-rings might be recondensed by precise titration.

Based on the qualitative appearance of the Z-rings, we chose 0.01 mM IPTG induction of ZapA as our standard condition for increasing Z-ring condensation in the *noc* mutant. Quantitatively, the FtsZ ring appeared to be condensed at either the midcell or cell pole in 89% of wild-type cells and in 49% of *noc* mutant cells (Fig. 6C**)**. Induction of ZapA in the *noc* mutant with 0.01 mM IPTG increased the percentage of cells that had a tightly condensed Z-ring to 78% (Fig. 6C). We observed that induced ZapA expression was sufficient to recondense and stabilize Z-rings in the *noc* mutant. Moreover, kymograph analysis indicated that when ZapA expression was induced in the *noc* mutant, the Z-rings appeared less mobile at the midcell and poles (Fig. 6D**;** Movie S7). We conclude that overexpression of the Z-ring-stabilizing protein ZapA can compensate for the absence of Noc and reduce Z-ring migration over the chromosome. We further conclude that the primary function of Noc is not to prevent *de novo* Z-ring formation over the chromosome but rather to function, like ZapA, in restricting Z-ring migration.

10.1128/mBio.02964-20.10 MOVIE S7 *noc* mutant containing an inducible construct of ZapA and fluorescent FtsZ grown in the microfluidic channel in the presence of 0.01 mM IPTG. Cell body (*P_sigA_-mRFPmars*) is false colored red, and mNeongreen-FtsZ is false colored green. Strain DK8144. Movies are 100 frames over 200 min at a rate of 1 frame/2 min. Download Movie S7, AVI file, 0.9 MB. Copyright © 2021 Yu et al. 2021 Yu et al. This content is distributed under the terms of the Creative Commons Attribution 4.0 International license.

## DISCUSSION

One of the primary topological determinants of bacterial cell division is the phenomenon of nucleoid occlusion, in which FtsZ rings are prevented from being superimposed with the bulk of the chromosome (9, 10, 13). In B. subtilis, nucleoid occlusion is thought to be mediated in part by the Noc protein, which, in turn, may directly or indirectly inhibit FtsZ dynamics. In support of an inhibitory role, the *noc* gene was originally identified as being synthetically lethal in the absence of the Min system, such that in a *min noc* double mutant, FtsZ polymerized in an unrestricted and unproductive manner throughout the cell (15). Moreover, overexpression of Noc exhibited a modest inhibition of cell division, which could be enhanced by the presence of a high-copy-number plasmid containing a Noc-binding sequence (15, 18). Noc binds preferentially to origin-proximal sites across the chromosome, but because the terminus remains at midcell after replication, the absence of Noc might act as a timer and/or cytokinetic guide for midcell Z-ring assembly (18, 43). If and how Noc functions as an inhibitor of FtsZ is unknown, but no specific or direct interaction between the two proteins has ever been reported, and current models suggest that the DNA-Noc-membrane supercomplex passively occludes FtsZ by steric obstruction (19). Here, we perform time-lapse video microscopy of FtsZ dynamics and show that Noc acts to corral FtsZ by preventing protofilaments from migrating away from the Z-ring during cytokinesis.

One role of nucleoid occlusion may be to control cell division site selection and direct FtsZ to form a condensed predivisional ring between the segregated nucleoids at the midcell. Noc might mediate nucleoid occlusion to guide FtsZ ring positioning, but work monitoring the first cell division event after spore germination showed that FtsZ formed a ring at the midcell between chromosomes with high fidelity, even in the absence of Noc (43). Our time-lapse observations and quantitative microscopy also argue against a role of Noc in cell division site selection under standard growth conditions. We found that the FtsZ ring formed in the middle of the bi-lobed nucleoid structure when the amount of chromosome material (indirectly indicated by the presence of the HBsu nucleoid binding protein) was spatially reduced to 60% of maximum intensity in both the wild type and the *noc* mutant (7). Thus, if occlusion by the nucleoid directs Z-ring localization, the occlusion somehow becomes abrogated when the local nucleoid volume is reduced by approximately half. By whatever mechanism nucleoid constriction is interpreted, Noc does not appear to be explicitly required (43).

**FIG 7:**
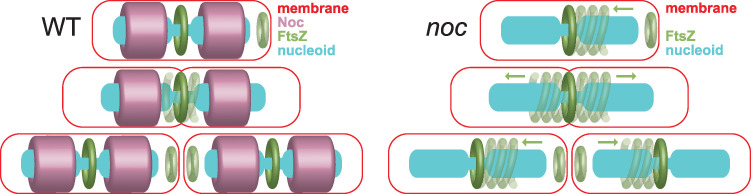
Cartoon model of the Noc corralling function. Noc binds to the chromosome and membrane and corrals migration of FtsZ protofilaments during cytokinesis. In the absence of Noc, traveling protofilaments depleted for ZapA travel from a condensed FtsZ focus toward the future site of cell division. FtsZ recondenses at the same position, in the interchromosome space where the nucleoid density is reduced regardless of whether Noc is present. Red, membrane; green, FtsZ; purple, Noc; cyan, nucleoid. Arrows indicate direction of travel of the decondensed Z-ring.

Another role of nucleoid occlusion may be to inhibit the *de novo* formation of FtsZ rings over the bulk of the nucleoid. Previous work showed that when Noc was mutated, decondensed helical rings of FtsZ were inappropriately superimposed over the nucleoid, consistent with the idea that Noc prevented FtsZ ring formation (15). Although we also observed superposition of decondensed helical FtsZ structures over the nucleoid, their superposition was not the result of *de novo* nucleation but rather of directional migration of an FtsZ subpopulation from the current cytokinetic Z-ring to the next site of cell division (Fig. 7). Thus, rather than preventing spontaneous Z-ring formation *per se*, Noc instead corralled and concentrated FtsZ at the location of an active Z-ring. A corralling mechanism may also explain the observation that FtsZ protofilaments are misaligned with the chromosome in *noc* mutants of Staphylococcus aureus, as FtsZ would migrate orthogonally from the division plane without topological restriction in a sphere (44, 45). In either organism, an extensive palisade of DNA-Noc-membrane complexes (19), previously thought to prevent *de novo* Z-ring formation, could instead form a steric impediment to prevent FtsZ migration over the chromosome and mediate the corralling phenomenon. We conclude that Noc promotes nucleoid occlusion by sterically impeding migration of FtsZ protofilaments from one Z-ring location to the next.

If Noc impedes the migration of decondensed FtsZ protofilaments, what governs FtsZ condensation into tight rings in the first place? Traveling FtsZ protofilaments are correlated with an elevated level of FtsZ (37, 46), but FtsZ levels do not appear to change in the absence of Noc. Instead, tight rings of FtsZ condensation colocalize with tight rings of the protofilament cross-linking protein ZapA, and decondensed traveling Z-foci in the *noc* mutant appear to be ZapA depleted. Consistent with local ZapA depletion, overexpression of ZapA recondenses the FtsZ foci into tight rings, but we infer that this effect is compensatory, because the levels of ZapA are, like that observed with FtsZ, unchanged in the *noc* mutant. We suggest that ZapA may be removed from a subpopulation of FtsZ protofilaments either stochastically or as a consequence of cytokinesis even in the wild type. Recent work suggests that the ZapA-FtsZ complex may oppose constriction, and accordingly, ZapA binding and removal may be a normal part of cytokinesis subject to spatiotemporal control (47). Thus, as cytokinesis proceeds, FtsZ protofilaments wander from the active Z-ring, and Noc may use the chromosome as a topological determinant to restrict FtsZ travel.

Our data support a role of Noc in the phenomenon of nucleoid occlusion but not for the purpose of protecting chromosome integrity or directing the location of the future site of cell division. Instead, the DNA-Noc-membrane supercomplex sterically restricts treadmilling protofilaments to the vicinity of the cytokinetic ring, after which they are depolymerized by the Min system for repolymerization at the next site of cell division. In the *noc* mutant, however, a subpopulation of protofilaments escapes the cytokinetic ring, travels over the chromosome, and arrives at the future site of cell division earlier than in the wild type. Nevertheless, the cell cycle is largely unperturbed, as Z-ring maturation requires a combination of *de novo* FtsZ synthesis and Min-mediated monomer recycling, and the rest of the division machinery relocates on time (27). Moreover, overexpression of ZapA compensated for the absence of Noc, perhaps by restricting FtsZ migration through increased protofilament cross-linking. We propose a model in which Noc increases division efficiency by forming a passive barrier that corrals and concentrates cell division machinery. We imagine that Noc functions in a manner analogous to eukaryotic septins that compartmentalize the cytokinesis of budding yeast and the bacterial dynamin homologs required for high-fidelity cell division during sporulation of Streptomyces coelicolor (28–31, 48).

## MATERIALS AND METHODS

### Strains and growth conditions.

Bacillus subtilis cells were grown in lysogeny broth (LB) (10 g tryptone, 5 g yeast extract, 10 g NaCl per liter) or on LB plates fortified with 1.5% Bacto agar at 37°C. Antibiotics were added when needed at the following concentrations: 100 μg/ml spectinomycin, 5 μg/ml kanamycin, 10 μg/ml tetracycline, 5 μg/ml chloramphenicol, and mls (macrolide-lincosamide-streptogramin B, 1 μg/ml erythromycin, 25 μg/ml lincomycin). For *P_hyspank_* promoter-dependent gene expression, 1 mM isopropyl-β-d-1-thiogalactopyranoside (IPTG) was added to the medium, if not otherwise indicated.

### Strain construction.

All strains were generated by direct transformation of DK1042, a derivative of B. subtilis ancestral strain 3610 with enhanced frequency of natural competence for DNA uptake, or were transduced into DK1042 by SPP1-mediated transduction (Table 1) (49, 50). Cells were mutated for the production of extracellular polysaccharide (EPS) to prevent biofilm formation within the microfluidic device by in-frame markerless deletion of *epsH* genes, encoding enzymes essential for EPS biosynthesis (51, 52). The mNeongreen fluorescent fusion to FtsZ or ZapA, a generous gift of Ethan Garner (Harvard University), was crossed into the indicated genetic background by SPP1 phage-mediated transduction, and the antibiotic resistance cassette was eliminated (32, 33). The HBsu-mCherry fusion (4, 34) was transduced into the indicated genetic backgrounds by SPP1 phage-mediated transduction. The *P_hyspank_-mCherry* inducible construct was introduced by integrating pEV6 at the *amyE* locus and selection for spectinomycin resistance (56).

**TABLE 1:** Strains

Strain	Genotype
DK1042	Wild type
DK3391	*amyE*::*P_hyspank_-mRFPmars spec*
DK5133	*mNeongreen-ftsZ ΔepsH amyE*::*P_hyspank_-mCherry spec*
DK5712	*mNeongreen-ftsZ ΔepsH sacA*::*hbsu-mCherry cat*
DK5820	*mNeongreen-ftsZ ΔepsH amyE*::*P_hyspank_-mcherry spec noc*::*kan*
DK6372	*mNeongreen-ftsZ ΔepsH sacA*::*hbsu-mCherry cat noc*::*kan*
DK8132	*amyE*::*P_hyspank_-zapA spec*
DK8138	*zapA-mNeongreen ΔepsH amyE*::*P_hyspank_-mcherry spec*
DK8139	*mNeongreen-ftsZ ΔepsH amyE*::*P_hyspank_-zapA spec ycgO*::*P_sigA_-mRFPmars mls*
DK8144	*mNeongreen-ftsZ ΔepsH amyE*::*P_hyspank_-zapA spec ycgO*::*P_sigA_-mRFPmars mls noc*::*kan*
DK8162	*mNeongreen-ftsZ ΔepsH ycgO*::*P_sigA_-mRFPmars mls*
DK8170	*mNeongreen-ftsZ ΔepsH ycgO*::*P_sigA_-mRFPmars mls noc*::*kan*
DK8172	*zapA-mNeongreen ΔepsH amyE*::*P_hyspank_-mcherry spec noc::kan*

### *noc*::*kan*.

The *noc*::*kan* insertion deletion allele was generated with a modified “Gibson” isothermal assembly protocol (53). Briefly, the region upstream of *noc* was PCR amplified with the primer pair 3399/3400, and the region downstream of *noc* was PCR amplified with the primer pair 3401/3402 (Table 2). DNA containing a kanamycin resistance gene (pDG780 [54]) was amplified with universal primers 3250/3251. The three DNA fragments were combined at equimolar amounts to a total volume of 5 μl and added to a 15-μl aliquot of prepared master mix (see below). The reaction mixture was incubated for 60 min at 50°C. The completed reaction was then PCR amplified with primers 3399/3402 to amplify the assembled product. The amplified product was transformed into competent cells of PY79 and then transferred to the 3610 background with SPP1-mediated generalized transduction. Insertions were verified by PCR amplification with primers 3399/3402.

**TABLE 2:** Primers

Primer	Sequence
3250	ACGACTCACTATAGGGCGAATTG
3251	CTCACTAAAGGGAACAAAAGCTGG
3399	TGCGACCGAGGCGCGTC
3400	CAATTCGCCCTATAGTGAGTCGTGAATGAATGCTTCATGTACCTAC
3401	CCAGCTTTTGTTCCCTTTAGTGAGCGCATACCAAAATAGAAGCTC
3402	AGGAATCACATCCAAGTTCTC
4572	AGGAGGCTAGCTGGAAGGAGGGATCCATAATGGCATCATCAGAAGATGTTA
4573	CTCCTGCATGCTTAGGATCCTGCACCTGTTGAATG
5266	TTTAAGGATCGTGTGATACGTGGAAGGAGGGATCCATAATGGCA
5267	TCTTCCCGATGATTAATTAATTAGGATCCTGCACCTGTTGAATGTC
5268	ATTATGGATCCCTCCTTCCACGTATCACACGATCCTTAAACCTAAAATTAATCAT
5269	TCACATTAATTGCGTTGCGCTATTTGATAGCCGAAGCGGAAAAAGCA
5270	CAACAGGTGCAGGATCCTAATTAATTAATCATCGGGAAGATCTTCATCACCGA
5271	TTTCTTCCTTTTCCTGTTTCAATTTATTCGTTTTATAAATGATTTTCCCGACATGGAAG
5272	TCCGCTTCGGCTATCAAATAGCGCAACGCAATTAATGTGAGTTAGG
5273	GTCGTTGCATTTTGTAAAGTGTCAGACTTTGATGAAGCTTTAGAAGTGGC
7278	TCAAAGCTTCGCGGAATGGAGGAGAAAC
7279	TCAGTCGACTCAATCCTTTTCTTTTAAGCTGAC

A 5× isothermal assembly reaction buffer (500 mM Tris-HCl [pH 7.5], 50 mM MgCl_2_, 50 mM dithiothreitol [DTT; Bio-Rad], 31.25 mM polyethylene glycol [PEG] 8000 [Fisher Scientific], 5.02 mM NAD [Sigma-Aldrich], and 1 mM each deoxynucleoside triphosphate [dNTP; New England BioLabs]) was aliquoted and stored at −80°C. An assembly master mixture was made by combining prepared 5× isothermal assembly reaction buffer (131 mM Tris-HCl, 13.1 mM MgCl_2_, 13.1 mM DTT, 8.21 mM PEG 8000, 1.32 mM NAD, and 0.26 mM each dNTP) with Phusion DNA polymerase (New England BioLabs; 0.033 U/μl), T5 exonuclease diluted 1:5 with 5× reaction buffer (New England BioLabs; 0.01 U/μl), *Taq* DNA ligase (New England BioLabs; 5,328 U/μl), and additional dNTPs (267 μM). The master mix was aliquoted as 15 μl and stored at −80°C.

### *amyE*::*P_hyspank_-mRFPmars*.

To generate the IPTG-inducible construct for mRFPmars expression, the gene encoding mRFPmars was PCR amplified from pmRFPmars (Addgene) with primers 4572/4573. The PCR product was purified, digested with NheI and SphI, and cloned into the NheI and SphI sites of pDR111 containing the *P_hyspank_* promoter, the gene conducing the LacI repressor protein, and an antibiotic resistance cassette between the arms of the *amyE* gene (generous gift from David Rudner, Harvard Medical School) to generate pDP430. The pDP430 plasmid was transformed into DK1042 to create DK3391.

### *ycgO*::*P_sigA_-mRFPmars*.

The constitutive mRFPmars construct was generated by isothermal assembly and direct transformation of the linear fragment into B. subtilis. The fragments upstream and downstream of *ycgO* were PCR amplified with primers 5270/5271 and 5272/5273, respectively, with chromosomal DNA from strain DK1042 as a template. The fragment containing the *P_sigA_* promoter was PCR amplified with primers 5268/5269 and chromosomal DNA from strain DK1042 as a template. The fragment containing mRFPmars and the spectinomycin resistance cassette was PCR amplified with primers 5266/5267 and chromosomal DNA from strain DK3391 as a template. The four PCR products were purified and mixed in an isothermal assembly reaction that was subsequently amplified by PCR with primers 5270/5273.

### *amyE*::*P_hyspank_-zapA spec*.

To generate the IPTG inducible construct for ZapA expression, the gene encoding ZapA was PCR amplified with primers 7278/7279 and chromosomal DNA from strain 3610 as a template. The PCR product was purified, digested with HindIII and SalI, and cloned into the HindIII and SalI sites of pDR111 to generate pYY8. The pYY8 plasmid was transformed into PY79 to create DK8132.

### Microfluidic system.

The microfluidic device was fabricated through a combination of electron beam lithography, contact photolithography, and polymer casting (26). Briefly, the microfluidic device is comprised of fluid and control layers both cast in poly(dimethylsiloxane) (PDMS) and a glass coverslip. The fluid layer lies between the control layer and glass coverslip and contains the microchannel array to trap the bacteria. Media and cells are pumped through the microfluidic channels by on-chip valves and peristaltic pumps that are controlled pneumatically through the top control layer. Each pneumatic valve is controlled by software to apply either vacuum (0.3 × 10^5^ Pa) or pressure (1.3 × 10^5^ Pa) to open or close individual valves, respectively.

### On-device cell culture.

Before cells were loaded into the microfluidic device, the microchannels were coated with 1% bovine serum albumin (BSA) in LB medium for 1 h to act as a passivation layer. Then, all the channels were filled with LB medium containing 0.1% BSA. A saturated culture of cells (∼10 μl) was added through the cell reservoir and pumped into the cell-trapping region. During cell loading, vacuum was applied to the control layer to lift up the microchannel array. After a sufficient number of cells were pumped underneath the channel array, positive pressure was applied to trap individual cells in those channels. Medium was pumped through the microchannels to flush away excess cells and maintain steady-state cell growth in the channel array.

### Time-lapse image acquisition.

After inoculation in the microfluidic channels, a period of roughly 3 h elapsed during which cells adjusted to the growth conditions, and steady-state cell growth was maintained and monitored over the next 21 h. Fluorescence microscopy was performed either on a Nikon Eclipse Ti-E microscope or an Olympus IX83 microscope. The Nikon Eclipse Ti-E microscope was equipped with a 100× Plan Apo lambda, phase-contrast, 1.45 numerical aperture (NA) oil immersion objective and a Photometrics Prime95B sCMOS camera with Nikon Elements software (Nikon, Inc.). Fluorescence signals from mCherry and mNeongreen were captured from a Lumencor SpectraX light engine with matched mCherry and yellow fluorescent protein (YFP) filter sets, respectively, from Chroma. The Olympus IX83 microscope was equipped with an Olympus UApo N 100×/1.49 oil objective and a Hamamatsu EM-CCD digital camera operated with MetaMorph Advanced software. Fluorescence signals from mCherry, mRFPmars, and mNeongreen were excited with an Olympus U-HGLGPS fluorescence light source with matched tetramethyl rhodamine isocyanate (TRITC), TRITC, and fluorescein isothiocyanate (FITC) filters, respectively, from Semrock. Images were captured from at least eight fields of view at 2-min intervals. The channel array was maintained at 37°C with a TC-1-100s temperature controller (Bioscience Tools). For all direct comparisons, the same microscope and settings were used.

### Data analysis.

An adaptation period following exposure to illumination was observed; thus, data analysis was restricted to periods of steady state. Cell identification and tracking were analyzed by MATLAB programs (The MathWorks, Inc.) (26). The program extracted fluorescence intensity along a line profile down the longitudinal center of each microchannel in the array. The cytoplasmic mCherry line profile showed a flat-topped peak on the line where a cell was located, and a local 20% decrease in fluorescence intensity was used to identify cell boundaries after division. Division events were conservatively measured as the time at which one cell became two according to the decrease in fluorescence intensity. Moreover, cell bodies were tracked from frame to frame in order to construct lineages of cell division, and cell body intensity was determined by the integration of the cytoplasmic mCherry signal intensity within the confines of the cell. Signals from HBsu-mCherry and mNeongreen-FtsZ were similarly tracked and measured along the length of the cell. FtsZ line profiles were normalized by cell body intensity in order to minimize intensity differences among frames and across different fields of view.

### Western blotting.

B. subtilis strains were grown in LB medium to an optical density at 600 nm (OD_600_) of ∼1.0, and 1 ml was harvested by centrifugation, resuspended to 10 OD_600_ in lysis buffer (20 mM Tris [pH 7.0], 10 mM EDTA, 1 mg/ml lysozyme, 10 μg/ml DNase I, 100 μg/ml RNase I, 1 mM phenylmethylsulfonyl fluoride [PMSF]), and incubated 30 min at 37°C. Ten microliters of lysate was mixed with 2 μl 6× SDS loading dye. Samples were separated by 15% sodium dodecyl sulfate-polyacrylamide gel electrophoresis (SDS-PAGE). The proteins were electroblotted onto nitrocellulose and developed with a 1:80,000 dilution of primary antibody (either anti-SigA or anti-FtsZ) and a 1:1,000 dilution secondary antibody (horseradish peroxidase [HRP]-conjugated goat anti-rabbit immunoglobulin G). Immunoblots were developed with the Immun-Star HRP developer kit (Bio-Rad).

### Microscopic image acquisition on agarose pads.

Cells were grown to mid-log phase in liquid culture and then imaged on 1% agarose pads in S750 medium (55). Fluorescence microscopy was performed with a Nikon 80i microscope with a Nikon Plan Apo 100× phase-contrast objective and an Excite 120 metal halide lamp. Cytoplasmic mRFPmars was visualized with a C-FL HYQ Texas Red filter cube, and mNeongreen-FtsZ was visualized with a C-FL HYQ FITC filter cube. Images were captured with a Photometrics Coolsnap HQ2 camera in black and white, false colored, and superimposed with Metamorph image software.
